# Effects of combo therapy with coenzyme Q10 and mitochondrial transplantation on myocardial ischemia/reperfusion-induced arrhythmias in aged rats 

**DOI:** 10.22038/ijbms.2024.80092.17348

**Published:** 2025

**Authors:** Soleyman Bafadam, Behnaz Mokhtari, Alireza Alihemmati, Reza Badalzadeh

**Affiliations:** 1 Drug Applied Research Center, Tabriz University of Medical Sciences, Tabriz, Iran; 2 Molecular Medicine Research Center, Biomedicine Institute, Tabriz University of Medical Sciences, Tabriz, Iran; 3 Department of Physiology, Faculty of Medicine, Tabriz University of Medical Sciences, Tabriz, Iran; 4 Department of Anatomical Sciences, Faculty of Medicine, Tabriz University of Medical Sciences, Tabriz, Iran

**Keywords:** Arrhythmia, Coenzyme Q_ 10_ Mitochondria, Myocardial ischemia/reperfusion injury, Oxidative stress

## Abstract

**Objective(s):**

Ischemia/reperfusion (IR)-induced ventricular arrhythmia, which mainly occurs after the opening of coronary artery occlusion, poses a clinical problem. This study aims to investigate the effectiveness of pretreatment with coenzyme Q_10_ (CoQ_10_) in combination with mitochondrial transplantation on IR-induced ventricular arrhythmias in aged rats.

**Materials and Methods:**

Myocardial IR induction was performed by left anterior descending coronary artery occlusion for 30 min, followed by re-opening for 24 hr. CoQ_10_ was administered intraperitoneally at a dosage of 10 mg/kg/day for two weeks before inducing IR. At the start of reperfusion, 500 µl of the respiration buffer containing 6×10^6^±5×10^5^ mitochondria/ml of respiration buffer harvested from the pectorals major muscle of young donor rats were injected intramyocardially. To investigate arrhythmias, the heart’s electrical activity during ischemia and the first 30 min of reperfusion were recorded by electrocardiogram. After 24 hr of reperfusion, cardiac histopathological changes, creatine kinase-MB, nitric oxide metabolites (NOx), oxidative stress markers (malondialdehyde, total anti-oxidant, superoxide dismutase, and glutathione peroxidase), and the expression of genes regulating mitochondrial fission/fusion were measured.

**Results:**

Pretreatment with CoQ_10_ in combination with mitochondrial transplantation reduced ventricular arrhythmias, cardiac histopathological changes, and creatine kinase-MB levels. Simultaneously, this combined therapeutic approach increased myocardial NOx levels, fostering an improved oxidative balance. It also triggered the down-regulation of mitochondrial fission genes, coupled with the up-regulation of mitochondrial fusion genes.

**Conclusion:**

The combination of CoQ_10 _and mitochondrial transplantation demonstrated a notable anti-arrhythmic effect by elevating NOx levels, reducing oxidative stress, and improving mitochondrial fission/fusion in aged rats with myocardial IRI.

## Introduction

Aging, recognized as a potential risk factor for cardiovascular disorders (CVD), entails a multifaceted process marked by the gradual decline of various physiological functions (1). This intricate aging process imparts several biological changes to cardiomyocytes, rendering the heart more susceptible to CVD (2, 3). Among age-related CVD, acute myocardial infarction (AMI) stands out as a significant cause of worldwide mortality and morbidity. Reperfusion therapy, a cornerstone in the management of AMI, aims to restore blood flow to the ischemic myocardium promptly. While reperfusion is essential for salvaging heart tissue and preventing extensive damage, paradoxically, it introduces a phenomenon known as myocardial ischemia/reperfusion injury (3, 4). Within the landscape of myocardial IRI, prominent concern emerges in the form of reperfusion-induced arrhythmias. These arrhythmias, ranging from ventricular tachycardia to potentially life-threatening ventricular fibrillation, can occur during or after the restoration of blood flow. Ventricular arrhythmias, in particular, can significantly impact patient outcomes and necessitate targeted interventions (5).

The aging heart is confronted with a complex interplay of factors that elevate the risk of arrhythmias during reperfusion following ischemic events, and central to this intricate scenario are mitochondrial dysfunction and oxidative stress (6). During the aging process, mitochondria experience alterations in both structure and function. These changes encompass a decline in integrity, dynamics, and respiratory chain activity, as well as deformities such as swelling and shrinkage, DNA mutations, and a rise in the production of reactive oxygen species (ROS) (7, 8). The age-related mitochondrial changes are exacerbated during the reperfusion phase, creating a conducive environment for the initiation and propagation of arrhythmias. Dysfunctional mitochondria contribute to disturbances in ion channel activity, disrupt cellular signaling pathways, and impair calcium handling, collectively fostering electrical instability in the aging myocardium (6, 9). Simultaneously, the delicate balance between ROS production and the anti-oxidant defense system is perturbed in the aging heart, leading to increased oxidative damage. This heightened oxidative stress further amplifies the risk of arrhythmias, creating a vicious cycle that exacerbates cardiac electrical instability (10). Interventions aimed at preserving mitochondrial function, reducing oxidative stress, and maintaining the delicate balance of cardiac electrophysiology hold promise for mitigating the heightened vulnerability to arrhythmias in the aging population during reperfusion events (11, 12)

Mitochondrial transplantation has emerged as a cutting-edge therapeutic strategy with promising implications in mitigating myocardial IRI (13, 14). Recent studies have demonstrated the efficacy of mitochondrial transplantation in enhancing cellular bioenergetics, reducing oxidative stress, and preserving cardiac function, making it a compelling avenue for exploring novel therapeutic interventions (13). Coenzyme Q_10_ (CoQ_10_) has garnered significant attention for its dual functions as a crucial component in mitochondrial bioenergetics and a potent anti-oxidant in mitigating the detrimental effects of reperfusion injury in the context of myocardial ischemia (15, 16). Previous studies have highlighted the cardioprotective effects of CoQ_10_, demonstrating its ability to reduce infarct size, preserve cardiac function, and mitigate arrhythmias following IRI (15, 17). In the context of aged IR myocardium, both mitochondrial function and CoQ_10_ levels exhibit a decline (18). Thus, combining CoQ_10_ supplementation with mitochondrial transplantation appears to be a valid approach, effectively addressing the concomitant age-related depletion in both aspects. This synergy may hold the potential to significantly enhance the overall efficacy of the intervention, potentially yielding more robust cardioprotection. Notably, this dual intervention may provide a comprehensive and synergistic approach to mitigate the multifaceted aspects of reperfusion injury, encompassing mitochondrial dysfunction, oxidative stress, and perturbations in cardiac electrophysiology.

In our previous work, we demonstrated that the combined strategy of mitochondrial transplantation and CoQ_10_ exerted cardioprotective effects, resulting in reduced cardiac infarct size and enhanced mitochondrial function (19). However, while these findings were promising, a crucial aspect of IRI that remained to be addressed was the management of life-threatening cardiac arrhythmias. The present study was designed to address this important gap in knowledge. Arrhythmias are one of the major complications associated with IRI, and their effective management holds significant clinical importance for both the prevention of electrical disturbances and cardioprotection. Therefore, the novelty and importance of the current investigation lie in its focus on evaluating the impact of the mitochondrial transplantation and CoQ_10_ combination therapy on preserving cardiac electrophysiology and reducing the risk of ventricular arrhythmias following IRI. Specifically, this study assesses the effects of combined treatment on ventricular arrhythmias, the expression of genes related to mitochondrial fission (Fis1; mitochondrial fission 1 protein, DRP; dynamin-related protein 1) and fusion (Mfn1; mitofusin 1, Mfn2; mitofusin 2), and oxidative stress in the aged rat heart exposed to IRI. By addressing both the bioenergetic aspects of mitochondrial health and myocardial oxidative stress in the context of aging, this investigation provides a comprehensive understanding of the anti-arrhythmic potential of this combination therapy, which represents a significant advancement beyond the previous cardioprotective findings.

## Materials and Methods

***Animals and ethics***

In this study, 48 old male Wistar rats (aged 22-24 months, weighing 450-500 g) and 10 young male Wistar rats (aged 6 weeks, weighing 200-250 g) were obtained from the animal center of Tabriz University of Medical Sciences (Tabriz, Iran). Animals were housed in standard cages in a temperature-controlled room (22±2°C) with a 12/12-hour light/dark cycle. They had ad libitum access to standard food and water. All animal procedures adhered to the National Institutes of Health (NIH) Guide for the Care and Use of Laboratory Animals (Publication No. 85-23, Revised 1985), and the Ethics Committee of Tabriz University of Medical Sciences approved all experimental protocols (Approval Numbers: IR.TBZMED.AEC.1401.065 and IR.TBZMED.REC. 1399.425).

***Experimental design***

Forty-eight rats were randomly allocated into six groups (n = 8/each group) as follows:

1) Sham: rats underwent thoracotomy without induction of IR; 

2) IR: rats were subjected to IR; 

3) CoQ_10_: rats received CoQ_10_ (Sigma, USA) at a dose of 10 mg/kg/day, intraperitoneally (IP), for two weeks and followed by IR (20);

4) MT: rats were subjected to IR and received 500 µl intramyocardial injection of 6 × 10^6^ ± 5 × 10^5^ mitochondria/ml of respiration buffer at 5 different sites around the infarcted area immediately at the onset of reperfusion (21); 

5) MT+CoQ_10_: rats received CoQ_10_ (10 mg/kg/day, IP) for two weeks and were subjected to IR. Additionally, they received isolated mitochondria via intramyocardial injection at the onset of reperfusion; and 

6) Vehicle: rats received 1% DMSO for two weeks and were subjected to IR. Additionally, they received 0.5 ml respiration buffer via intramyocardial injection immediately at the onset of reperfusion. 

In our previous study (19), doses of interventions were selected based on a pilot dose-response study and the existing literature showing their significant impact on tissue protection. The current study continues with the same doses identified in the earlier research to further investigate their impact. After the experimental procedures, cardiac blood samples were collected under anesthesia, and following centrifugation, sera were separated and stored at -80 °C until creatine kinase-MB (CK-MB) measurement. Subsequently, the rats were sacrificed under deep anesthesia, and their hearts were immediately excised. In each group, the left ventricles of three hearts were fixed for histopathological assessment. Left ventricular anterior wall tissue from the remaining five hearts in each group was dissected and stored at -80 °C for subsequent analysis. As no significant differences were observed between the vehicle and IR groups, data from the vehicle group were not included in the article.

***Mitochondria isolation***

**Figure 1:**
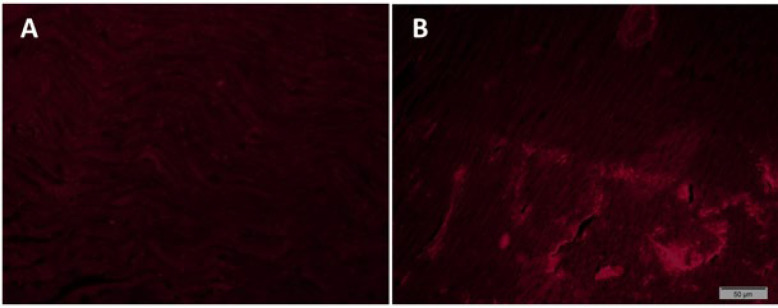
Fluorescent microscope imaging of cardiac tissue of aged rats

Healthy young male rats were used as mitochondrial donors. After anesthesia with ketamine and xylazine (at a ratio of 80:10 mg/kg), a piece of pectoralis major muscle (0.21±0.06 g wet weight) was removed from the animals and instantly harvested in mitochondrial isolation buffer at 4 °C, comprising: 200 mM mannitol, 70 mM sucrose, 10 mM 4-(2-hydroxyethyl)-1-piperazineethanesulfonic acid (HEPES), and 2 mM ethylenediaminetetraacetic acid (EDTA), pH 7.5. The homogenate was centrifuged for 10 minutes at 1200 *g* at 4 °C. The supernatant was collected and centrifuged again at 12000 g for 10 minutes at 4°C. Finally, the supernatant was discarded and the pellet was resuspended in 100 µl of storage buffer comprising: 1 mM ATP, 250 mM sucrose, 10 Mm HEPES, 1 mM dithiothreitol, 5 mM sodium succinate, 0.08 mM adenosine diphosphate, and 2 mM K2HPO4 (22). The isolated mitochondria were counted by hemocytometry method, and finally, 6 × 10^6^ ± 5 × 10^5^ mitochondria/ml of respiration buffer, dissolved in 0.5 ml respiration buffer, were injected into the left ventricle (LV) of the MT receiving groups. To ensure the effective absorption and integration of mitochondria into the cardiac tissue via intramyocardial injection, in a primary pilot investigation, the isolated mitochondria were incubated with a concentration of 50 ng/ml of MitoTracker^TM^ RED (Invitrogen, USA) at 37 °C. Subsequently, the labeled mitochondria with MitoTracker^TM^ RED were injected to the LV. Twenty-four hours post-injection, the heart was extracted, and tissue sections were prepared for examination of the labeled mitochondria using a fluorescence microscope. Remarkably, it was observed that the labeled mitochondria had effectively entered the cardiac tissue (Figure 1). The heart tissue that received Mitotracker RED-labeled mitochondria emitted red fluorescence under the fluorescence microscope, while the heart tissue that did not contain the labeled mitochondria showed no red fluorescence.

***Myocardial IRI modeling***

The myocardial IR model was established in rats based on a previously described model briefly, aged rats were anaesthetized with ketamine and xylazine (at a ratio of 80:10 mg/kg, IP) and ventilated with a rodent mechanical ventilator after intratracheal cannulation. The animal was restrained in the supine position on a heating pad (temperature 37 ± 1 °C) to maintain the animal’s physiological body temperature. To expose the animal’s heart, a left parasternal incision was made through the third intercostal space and the pericardium was opened. Regional ischemia was induced by clamping the Left Anterior Descending coronary artery (23) using a 6-0 suture 2 to 3 mm below the left atrial appendage for 30 minutes. For reperfusion, the LAD clamp was released and the chest closed and sutured. 

***Electrocardiogram (ECG) and arrhythmias interpretation***

**Figure 2:**
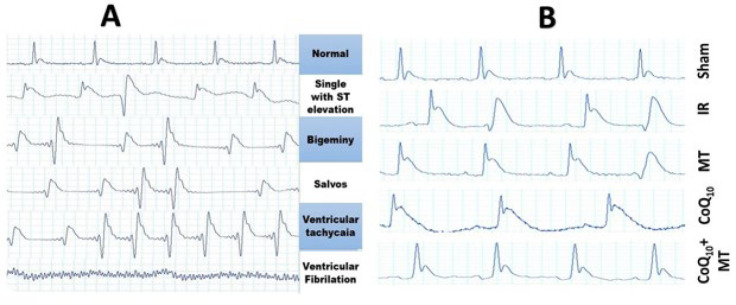
(A) Electrocardiogram recordings displaying different types of ventricular arrhythmias as per the Lambeth Convention and (B) Sample ECG tracing in experimental groups of aged rats

Electrical activity of the heart was monitored by ECG, recorded during 30 minutes of ischemia and the first 30 minutes of reperfusion, digitized via a data collection system (AD Instruments, Australia), and analyzed by LabChart v7.7 (AD Instruments, Newcastle, NSW, Australia). Ventricular arrhythmias were classified according to the Lambeth Conventions (24), categorized as single ventricular premature beats (VPB), ventricular bigeminy (VB), ventricular salvos (VS), ventricular tachycardia (VT), and ventricular fibrillation (VF) (Figure 2A). Figure 2B shows the sample ECG tracing of different groups of experimental animals. The sum of VPB, VB, and VS is termed premature ventricular contraction (PVC) (25). The ECG of each rat was analyzed for 60 minutes to evaluate total PVC, duration of VT and/or VF. The scoring of arrhythmias in each rat was performed based on a 5-grade evaluation system (Table 1) (26): grade 0; no arrhythmia, grade 1; VPB, grade 2; VB or VS, grade 3; VT, and grade 4; VF. For the sample with more than one type of arrhythmia, the higher grade was reported (25). 

***Histopathology assay***

Left ventricular tissue from animals was isolated and fixed in formalin 10% for 48 hr. After processing, paraffin embedded blocks were prepared. The blocks then were sectioned in 5 *μ*M thickness and stained by hematoxylin and eosin (H&E) to examine histopathological changes. Illustrative photomicrographs were taken under a light microscope equipped with a camera and histological alterations including edema, vacuolization of cytoplasm, marginal and pyknotic nuclei, myocyte degeneration or necrosis and leukocyte infiltration were quantified through ordinal scoring of the severity of histopathologic changes (zero to 4 scores) (27). Five fields were randomly selected for examination in each sample and scored as follow: zero; absence of pathological changes in these fields (normal), 1; changes in only one field (minimal), 2; changes in two fields (mild). 3; changes in three fields (moderate), 4; changes in more than three fields (sever).

***Measurement of CK- MB activity***

CK-MB activity was measured in serum samples by the colorimetric method according to the protocol provided by the kit supplier (Biorexfars, Iran, cat No; Nx2332; BXC0452) and presented as U/l.

***Measurement of oxidative stress markers***

To assess the oxidative level in the cardiac tissue of all groups, 100 mg of frozen LV tissue was homogenized in phosphate buffer solute (containing EDTA). The levels of malondialdehyde (MDA) and total anti-oxidant status (TAS), as well as the enzyme activities of superoxide dismutase (SOD) and glutathione peroxidase (GPx), were measured in the supernatant of the homogenate.

***MDA***

MDA level, as a marker of lipid peroxidation, was measured based on the thiobarbituric acid (TBA) method (28). According to this method, 1 ml of supernatant was blended with 2 ml of trichloroacetic acid –TBA - hydrochloric acid and the solution was heated for 40 minutes in a boiling water bath under acidic conditions and high temperature, MDA conjugates with TBA and forms a purple complex of MDA-TBA with maximum absorption at 535 nm; after cooling, the supernatant of the mixture was aliquoted and its absorbance was measured at 535 nm. MDA level was expressed as nmol/mg protein.

***TAS***

TAS was evaluated using a commercial kit (Randox, UK, cat No; Nx2332) according to the manufacturer’s instructions and results were presented as μmol/mg protein.

***SOD.*** SOD activity was evaluated using a commercial kit (Randox, UK, cat No; SD125) as per the manufacturer’s instructions. Xanthine and xanthine oxidase produce superoxide radicals which react with 2-(4-iodophenyl)-3-(4-nitrophenol)-5-phenyltetrazolium chloride to produce a formazan dye. SOD activity was evaluated based on the reduction of color formation, measured at 440 nm, and reported as U/mg protein.

***GPx***

GPx activity in the supernatant was assayed using a commercial kit (Randox, UK, cat No; RS504), according to the manufacturer’s instructions. GPx converts oxidized glutathione to its reduced form in the presence of NADPH and glutathione reductase, accompanied by the oxidation of NADPH to NADP^+^ and a reduction in NADPH absorbance. The decline in supernatant absorbance at 340 nm was measured and the enzyme activity was reported as nmol/min/mg protein.

***Measurement of NO metabolites (NOx)***

The Griess method was used to evaluate NOx contents in LV tissue samples (29). Briefly, samples were homogenized in phosphate-buffered saline and centrifuged at 12,000g for 10 minutes at 4°C. The supernatants were collected and deproteinized by addition of zinc sulphate (15 mg/ml). A 100 μl supernatant and 100 μl vanadium (III) chloride (8 mg/ml) were added to a microplate well (for reduction of nitrate to nitrite), after that 50 μl sulfanilamide (2%, dissolved in chloridoid acid 5%) and 50 μl (N-(1-naphtyl) ethylenediamine dihydrochloride , dissolved in chloridoid acid 5%) were added and after 30 minutes incubation at 37 °C, the absorbance was read at 540 nm The concentration of NOx in the samples was measured from the linear standard curve established by 0-50 μmol/l of sodium nitrate and presented as μmol/g protein.

***Assessment of gene expression***

A piece of LV area at risk was used for total mRNA extraction by an RNA isolation kit (Yekta Tajhiz Azma, Iran) following the manufacturer’s instructions. The extracted RNA was quantified by Thermo Scientific Nanodrop 1000 spectrophotometer and 1000 ng of RNA was used for reverse transcription reaction to synthesize complementary DNA (cDNA) using cDNA Synthesis Kit (Yekta Tajhiz Azma, Iran). The relative RNA expression of Fis1, Drp1, Mfn1, Mfn2, and GAPDH genes was evaluated by real-time PCR method using SYBER Green master mix (Yekta Tajhiz Azma, Iran) and QPCR System (Rotor-Gene RG-6000 Real-Time PCR Analyzer (Corbett, Australia)). GAPDH was used as the internal control. Relative RNA expression of target genes was calculated using the 2^−ΔΔCT^ method (30) and reported as fold changes to the sham group. Primers used are listed in Table 2.

***Statistical analysis***

Data were expressed as mean ± SEM. The non-parametric Kruskal-Wallis test was used to analyze data of the myocardial arrhythmia and histological findings between groups, and the parametric test of one-way ANOVA followed by Tukey’s post hoc test was used to compare differences in other parameters including; CK-MB, oxidative stress markers, NOx, and relative gene expression between groups. In all cases,* P*<0.05 was considered statistically significant.

## Results

***Myocardial arrhythmias***

**Figure 3:**
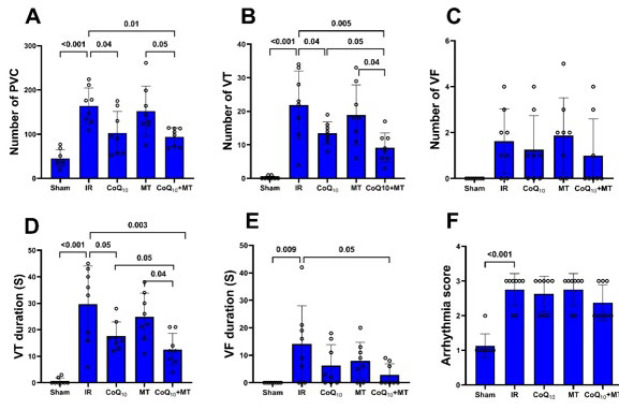
Ventricular arrhythmias through 30 min of ischemia and the first 30 min of reperfusion in experimental groups of aged rats

Figure 3 indicates the number and duration of different ventricular arrhythmias including PVCs, VTs, and VFs as well as the arrhythmia score in experimental groups. The results show that the number of PVCs and VTs in the IR group was significantly higher compared to the sham group (*P*<0.001 for both), while there was no significant difference in the number of VFs between the groups. The number of PVCs and VTs in the CoQ_10_ (*P*<0.05) and CoQ_10_+MT (*P*<0.01) groups was significantly lower than in the IR group. The number of PVCs in CoQ_10_+MT group was significantly lower than MT group (*P*<0.05). Additionally, the CoQ_10_+MT treatment resulted in a significantly greater reduction in the number of VTs compared to the individual treatments of CoQ_10_ and MT (*P*<0.05 for both). Moreover, the IR group exhibited a significantly longer duration of VT (*P*<0.001) and VF (*P*<0.01) compared to the sham group. CoQ_10_ alone only reduced the duration of VT (*P*<0.05), but when administered along with MT, it reduced both the duration of VT (*P*<0.01) and VF (*P*<0.05) compared to the IR group. In addition, VT duration in the CoQ_10_+MT group was significantly shorter than in the CoQ_10_ and MT groups (*P*<0.05 for both). 

***Histological results***

**Figure 4:**
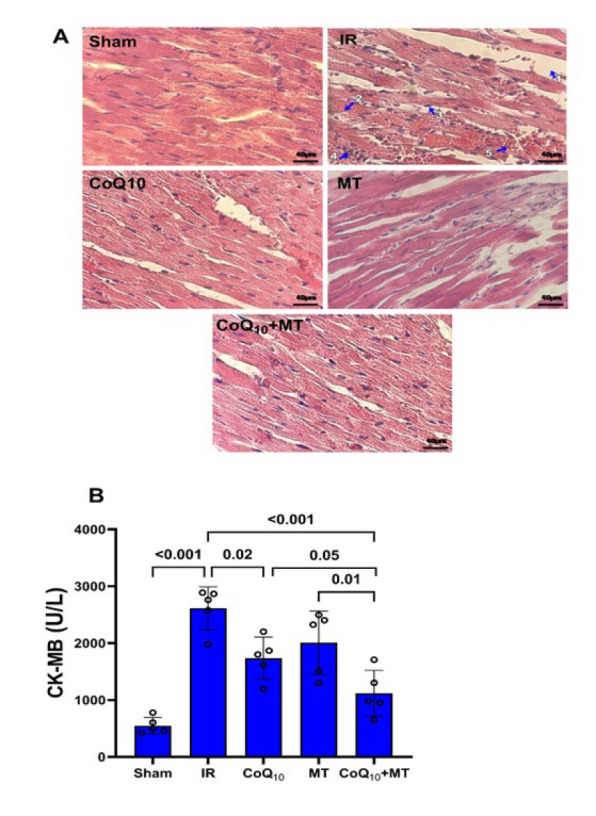
(A) Hematoxylin and eosin staining of heart tissue sections in different experimental groups of aged rats (n = 3 in each group). The blue arrows represent: 1. edema; 2. sarcoplasmic vacuolization; 3. marginal nuclei; 4. leukocyte infiltration; 5. myocyte degeneration. (B) Serum CK-MB levels in experimental groups (n = 5 in each group)

Figure 4 A shows the LV histological changes in experimental groups and their descriptive values are shown in Table 3. The sham group indicated normal cardiac tissue with arranged muscle fibers, and clear nuclear and muscle bands, conversely, the normal tissue structure in IR group was lost along with reduced muscle fibers organization, increased distance between cardiomyocytes (sign of edema), leukocytes infiltration, marginal and pyknotic nuclei, cytoplasm vacuolation, and degeneration and necrosis of cardiomyocytes. However, pretreatment with CoQ_10_ alone or in combination with MT alleviate those histopathological changes. The histopathological alterations were scored as outlined in the Methods section. As indicated in Table 3, the IR group exhibited significantly higher levels of pathological indicators, including edema, sarcoplasmic vacuolization, marginal nuclei leukocyte infiltration, and myocyte degeneration, in comparison to the sham group (*P*<0.001). Q1 treatment alone and in combination with MT, demonstrated a significant reduction in edema compared to the IR group (*P*<0.001 for both). Furthermore, the CoQ_10_+MT group exhibited a significantly lower amount of edema compared to the MT group (*P*<0.01). Sarcoplasmic vacuolization in CoQ_10_+MT group was significantly lower than IR, CoQ_10_, and MT groups (*P*<0.001, *P*<0.05 and *P*<0.01, respectively). The administration of CoQ_10 _alone and in combination with MT exhibited a significant decrease in marginal nuclei compared to the IR group (*P*< 0.05 and *P*<0.001, respectively). Moreover, the combined treatment group displayed a significantly lower level of this indicator compared to the CoQ_10 _and MT groups (*P*< 0.05 and *P*<0.001, respectively). The CoQ_10_+MT group exhibited a significant reduction in leukocyte infiltration compared to the IR and MT groups (*P*<0.05 and *P*<0.01, respectively). Although a reduction in leukocyte infiltration was observed in the single treatment group compared to the IR group, the difference was not found to be statistically significant. Furthermore, myocyte degeneration in the CoQ_10_ and CoQ_10_+MT groups exhibited a significant decrease compared to the IR group (*P*<0.05 and *P*<0.001 respectively). Additionally, the combined treatment group demonstrated a significantly lower level of myocyte degeneration compared to the MT group (*P*<0.01).

***Serum level of CK-MB activity***

Serum CK-MB activity as an indicator of cardiac damage in the IR group was significantly higher than those in the sham group (*P*<001). However, pretreatment with CoQ_10_ alone or in combination with MT significantly lowered this increase compared to the untreated IR group (*P*< .05 and *P*< 0.001 respectively). Furthermore, the CoQ_10_+MT treatment demonstrated a significantly lower value for this marker in comparison to the individual treatments with Q_10_ and MT (*P*<0.05 and *P*<0.001 respectively). (Figure 4B).

***Oxidative stress markers***

**Figure 5:**
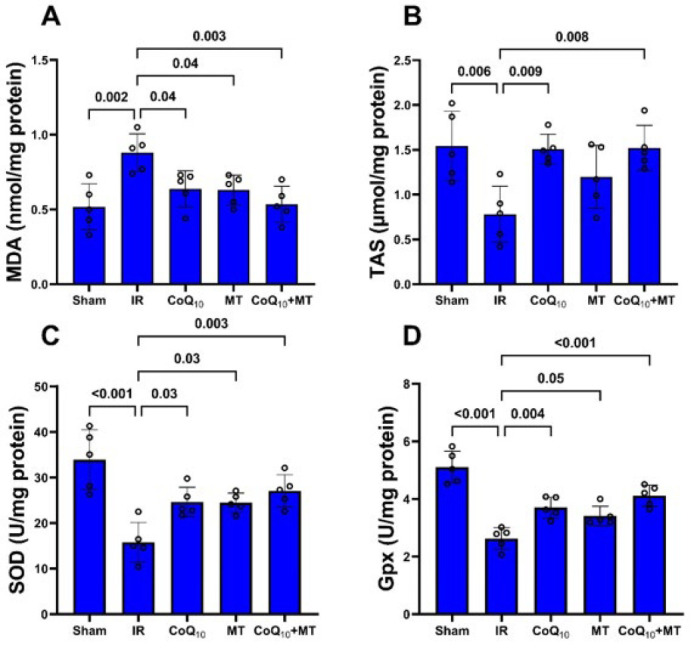
Oxidative stress markers in cardiac tissue samples of experimental groups of aged rats

Heart tissue MDA level in the IR group was significantly higher than in the sham group (P<0.01), whereas TAS was significantly lower in this group compared to the sham group (P<0.01). Pretreatment with CoQ_10_ alone and MT alone or in combination together significantly decreased heart tissue MDA level compared to the untreated IR group (P<0.05). Moreover, CoQ_10_ Pretreatment alone or in combination with MT significantly increased TAS in comparison with the untreated IR group (P<0.01), but although MT increased the TAS level to some extent, it was not statistically significant (Figure 5A and B). The activity of SOD and Gpx enzymes in the heart tissue of the IR group were significantly lower than sham group (P<0.001 for both), however, SOD activity in CoQ_10_, MT, and combination therapy groups was significantly higher than in the IR group (P<0.05). In addition, Gpx enzyme activity in CoQ_10_, MT, and combination therapy groups was significantly at a higher level compared to the IR group (P<0.01, P<0.05, P<0.001 respectively). (Figure 5C and D).

***Heart tissue nitrite/nitrate oxide (NOx) level***

**Figure 6:**
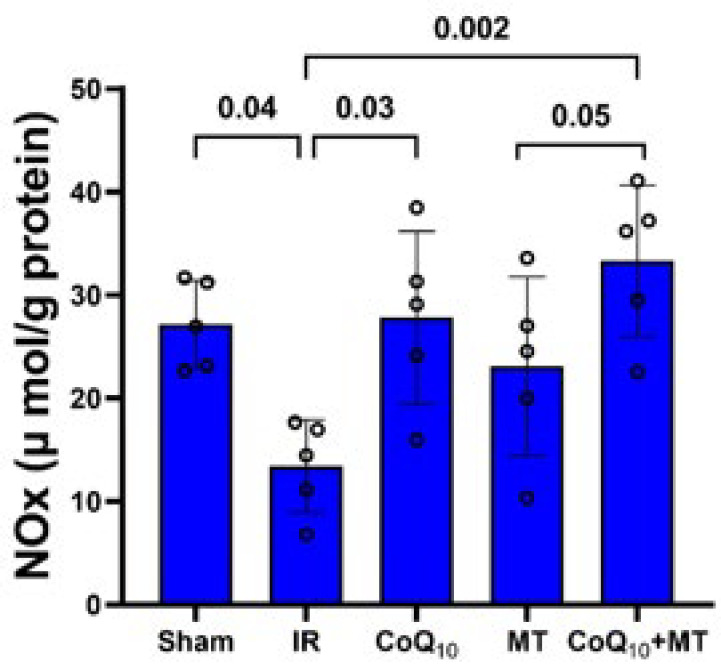
Cardiac tissue NOx levels in experimental groups of aged rats (n = 5 in each group)

The heart tissue NOx level in the IR group was significantly lower than the sham group (*P *<0.05). However, CoQ_10_ alone or in combination with MT significantly increased NOx level compared with the IR group (*P*<0.05) but MT alone could not significantly increase NOx level in comparison with the IR group. Additionally, the level of NOx in the CoQ_10_+MT group was significantly higher than in the MT group (*P*<0.05) (Figure 6). 

***Gene expression***

**Figure 7:**
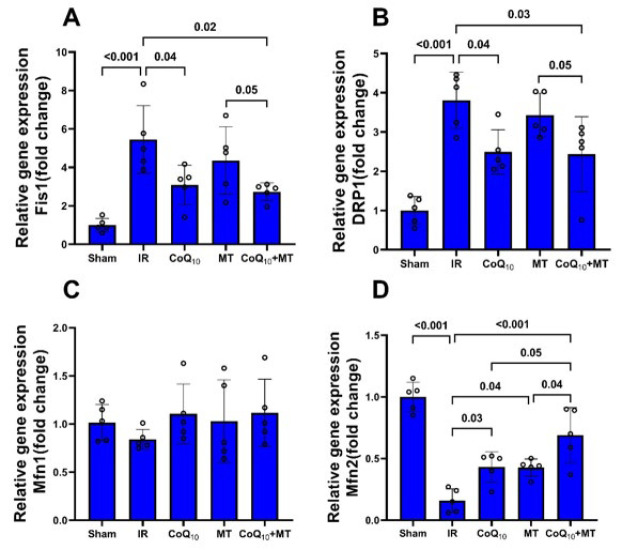
Relative gene expression of (A) Fis1, (B) DRP1, (C) Mfn1, and (D) Mfn2 in experimental groups of aged rats (n = 5 in each group)

The results of real-time PCR showed that left ventricle relative gene expression of Fis1 and DRP1 in the IR group were significantly greater than in the sham group (P<0.001), while the relative gene expression of Mfn2 in IR group was significantly lower than sham group (*P*<0.05), and also some decrease in Mfn1 relative gene expression was observed in the IR group as compared to sham group, but it was not statistically significant. Pretreatment with CoQ_10_ alone or in combination with MT significantly attenuated Fis1 and DRP1 relative gene expressions as compared with the untreated IR group (P<0.05). Relative expression of Fis1 and DRP1 genes in the MT group also decreased in comparison to the IR group, however, this reduction was not statistically significant. Furthermore, the relative gene expression of Fis1 and DRP1 in the CoQ_10_+MT group were significantly lower than in the MT group (*P*<0.05). The relative gene expression of Mfn2 in all treatment groups was significantly higher than in the IR group (*P*<0.05 and *P*<0.001), however its expression in the combination therapy group was significantly higher than in the single treatment groups (*P*<0.05). Moreover, Mfn1 relative gene expression in all treatment groups was higher than in the IR group but not statistically significant. (Figure 7). 

(A) without and (B) with mitochondrial transplantation, where the red fluorescence indicates Mitotracker RED-labeled mitochondria that have been transplanted into the cardiac tissue.

ECG: Electrocardiogram

**Table 1:** Scoring of arrhythmias based on the 5-grade evaluation system (25)

**Type of arrhythmia**	**Score**
**no arrhythmia**	0
**ventricular premature beats, VPB**	1
**ventricular bigeminy, VB; or ventricular salvos, VS**	2
**ventricular tachycardia, VT**	3
**ventricular fibrillation, VF**	4

VPB: Ventricular premature beats; VB: Ventricular bigeminy; VS: Ventricular salvos; VT: Ventricular tachycardia; VF: Ventricular fibrillation

**Table 2:** The primers sequence used in this study

Reverse primers sequence	Forward primers sequence	Gene
AGGCACCAGGCGTATTCAAA	GGGTTACATGGATGCCCAGA	Fis1
GAAATTTACCCCATTCTTCTGCT	TTCTTCCCAGAGGGACTGGT	Drp1
GCCAAAAAATGCCACTTTCATATGC	AAGCAACATACAGGAACCCGGAA	Mfn1-rn
CATCACAATGCCAGACACCAAC	CCTGGGCTTTAGACTCAACCAG	Mfn2-rn
CTTGCCGTGGGTAGAGTCAT	AGACAGCCGCATCTTCTTGT	GAPDH

Fis1: Mitochondrial fission 1 protein; DRP1: Dynamin-related protein 1; Mfn1: Mitofusin 1; Mfn2: Mitofusin 2

(A) Number of PVCs, (B) Number of VTs, (C) Number of VFs, (D) Duration of VT, (E) Duration of VF, and (F) Arrhythmia score in experimental groups (n = 8 in each group). All data were expressed as Mean ± SEM. IR: Ischemia/reperfusion; CoQ_10_: Coenzyme Q_10_; MT: Mitochondrial transplantation; PVC: Premature ventricular contraction; VT: Ventricular tachycardia; VF: Ventricular fibrillation.

All data were expressed as Mean ± SEM. IR: Ischemia/reperfusion; CoQ_10_: Coenzyme Q_10_; MT: Mitochondrial transplantation; CK-MB: Creatine kinase MB

**Table 3:** Degree of histopathological changes in myocardial tissue of different groups of aged rats

**Group**	**Edema**	**Sarcoplasmic vacuolization**	**Marginal nuclei**	**Leukocyte infiltration**	**Myocyte degeneration**
**Sham**	1.11±0.61	1.78±0.83	1.22±0.44	1.33±0.71	0.89±0.33
**IR**	3.77±0.44^***^	3.67±0.50^***^	3.89±0.33^***^	3.33±1.32^***^	3.67±1.00^***^
**CoQ**_10_	2.22±.67^###^	3.11±1.05^$^	3.00±0.87^#, $^	2.22±0.67	2.33±1.00^#^
**MT**	3.00±0.71^$$^	3.56±1.01^$$^	3.67±0.71^$$$^	2.89±0.78^$^	3.11±1.05^$$^
**CoQ**_10 _ **+ MT**	1.89±0.60^###^	1.93±0.74^###^	2.11±0.78^###^	1.56±1.13^##^	1.56±0.88^###^

Edema: increase in the distance between cardiomyocytes

Sarcoplasmic vacuolization: formation of fluid-containing vacuoles surrounded by myofibrils in the sarcoplasm

Marginal nuclei: Nuclei located far from the center of cardiomyocytes

Leukocyte infiltration: Migration of immune cells into the myocardial tissue

Myocyte degeneration: Necrotic death of cardiomyocytes

***P<0.001 vs Sham group. # *P*<0.05, ##*P* < 0.01 , and ### *P*<0.001 vs IR group. $*P*<0 .05, $$ *P*<0 .01, and $$$ *P*<0 .001 vs MT CoQ10+MT group.

The levels of (A) MDA and (B) TAS, and the activities of (C) SOD and (D) Gpx in experimental groups (n = 5 in each group). All data were expressed as Mean ± SEM.

IR: ischemia/reperfusion; CoQ_10_: Coenzyme Q_10_; MT: Mitochondrial transplantation; MDA: Malondialdehyde; TAS: Total antioxidant status; SOD: Superoxide dismutase; Gpx: Glutathione peroxidase

All data were expressed as Mean ± SEM. IR: Ischemia/reperfusion; CoQ_10_: Coenzyme Q_10_; MT: Mitochondrial transplantation; NOx: Nitrite/nitrate oxide

All data were expressed as Mean ± SEM.

IR: Ischemia/reperfusion; CoQ_10_: Coenzyme Q_10_; MT: Mitochondrial transplantation; Fis1: Mitochondrial fission 1 protein; DRP1: Dynamin-related protein 1; Mfn1: Mitofusin 1; Mfn2: Mitofusin 2.

## Discussion

The present study investigated the combined effect of CoQ_10_ and mitochondrial transplantation on IR-induced ventricular arrhythmias and cardioprotection in an *in vivo* model of aged rats. Pretreatment with CoQ_10_ was found to be able to reduce ventricular arrhythmias and histopathological changes, whereas MT alone did not produce significant beneficial effects. Notably, co-administration of CoQ_10_ and MT resulted in a greater reduction in ventricular arrhythmias and histopathological changes than CoQ_10_ alone. The cardioprotective and anti-arrhythmic effects of this combined treatment were associated with a reduction in cardiac oxidative stress, an increase in cardiac NOx levels and an improvement in the expression of mitochondrial fission/fusion-related genes in aged rats. 

Cardiovascular diseases, particularly coronary heart disease and myocardial infarction, mainly occur in the elderly, and the aging process is associated with a variety of detrimental biological changes in cardiomyocytes, including increased generation of ROS and subsequent oxidative stress, impaired autophagy, dysfunctional mitochondria, inflammation, impaired metabolic regulation, and ultimately cellular demise and death (2, 3, 31). Moreover, the presence of damaged and dysfunctional mitochondria in cardiomyocytes is a critical factor in the development and progression of cardiac aging (6). These adverse alterations can increase the vulnerability of the heart to IRI, thereby reducing the efficacy of individual therapeutic interventions in the context of aging. Hence, it is important to examine whether implementing a multi-intervention strategy could provide substantial protective benefits in aged hearts.

As the data reveals, CoQ_10_ indicated some positive effects against IR-induced arrhythmias and myocardial histopathological changes. Consistent with our results, Pagan *et al*. indicated an anti-arrhythmic effect of CoQ_10_ (17) and Liang *et al.* reported that preconditioning with CoQ_10_ attenuated myocardial IR-induced histopathological changes as indicated by reduction in myocardial cell denaturation and necrosis and delay in myocardial fibrosis (15) However, the observed effects of CoQ_10_ in the present study were not as reliable and robust as those of co-combination therapy. Specifically, in the combination therapy group, all the examined parameters showed an improvement trend, while in the CoQ_10_ group, some parameters did not show significant improvement. CoQ_10_ is one of the mitochondrial substances (18), so it is expected that its effects appear in part by interacting with the cardiac cell’s mitochondria. However, this study used old rats, which known to have defective and dysfunctional mitochondria due to the natural process of aging (18). Consequently, the administered CoQ_10_ interacts with these defective mitochondria, hindering its ability to effectively exert its protective effects. This highlights the importance of considering combination therapies in the context of aging. Hence, it is logical to suggest that if healthy mitochondria transplanted into the hearts of elderly rats suffering from IRI could potentially enhance the protective effects of CoQ_10_ through a synergistic or additive interaction and the stronger effects of combination therapy found in this study may support this hypothesis.

Numerous studies have investigated the potential of mitochondrial transplantation as a therapeutic approach to mitigate IRI, and in most cases the results have been promising (13, 14). However, in the present study, the effects of mitochondrial transplantation on the reduction of arrhythmias and cardioprotection were not statistically significant. One possible explanation for this discrepancy is that previous studies have mainly focused on young animal models without risk factors, whereas this study used aged rats to more closely simulate clinical conditions. It is well known that aging is associated with physiological and homeostatic changes in cardiomyocytes including a decrease in mitochondrial stimulators or enhancers such as CoQ_10_ (32). As a result, transplanted mitochondria may find it difficult to function optimally in this disturbed environment. It is noteworthy that in the combination therapy group, pre-treatment with CoQ_10_ replenishes intracellular CoQ_10_ reserves and may reinstate the intracellular environment, allowing healthy mitochondria to operate under more favorable conditions and exert their therapeutic potential. 

To elucidate the possible protective mechanisms of interventions given the increased oxidative stress, NO depletion and impaired mitochondrial dynamics associated with aging and IR, the changes of these mediators were assessed in left ventricular tissue. Oxidative stress is characterized by membrane damage, lipid peroxidation, protein conformational changes and ion channel dysfunction. It impairs membrane permeability and leads to electrolyte imbalance and calcium overload, culminating in ventricular arrhythmias (9, 10). One target of ROS attack is mitochondria, resulting in mitochondrial permeability transition pore opening, mitochondrial integrity disruption and respiratory complex dysfunction, reducing ATP production and exacerbating oxidative stress. The normal function of the ATP-dependent pump is disrupted as a result of ATP reduction. This exacerbates electrolyte imbalance and increases the incidence of arrhythmias (33, 34). In this study, the combined intervention significantly and remarkably reduced oxidative stress. This suggests a potential anti-arrhythmic mechanism through modulation of oxidative stress. This is consistent with previous findings demonstrating the anti-arrhythmic effects of well-known anti-oxidants such as vitamin E (35), resveratrol (36), and quercetin (37). 

Furthermore, NO is a mediator with diverse effects on IRI and cardioprotection and plays a crucial role in modulating the opening of the mitochondrial ATP-sensitive potassium channel (mitoK-ATP) (38). Previous studies have highlighted the cardioprotective effects of mitoK-ATP, indicating that NO-mediated mitoK-ATP activation enhances mitochondrial homeostasis and function and decreases ROS production (39). Additionally, the ability of NO to modulate the activity of the respiratory chain and to act as a scavenger of oxygen radicals further supports its potential to ameliorate oxidative stress and to reduce cardiac arrhythmias (39, 40). Consistent with these findings, our study demonstrated reduced arrhythmias in groups with elevated NOx (an indirect measure of NO) levels, suggesting improved mitochondrial conditions through reduced oxidative stress and increased NO levels. Improvement in mitochondrial integrity and function are expected to have a positive impact on mitochondrial dynamic mechanisms such as fission and fusion during IRI (41). The observed decrease in the expression of fission-related genes (Fis1, DRP1) and increase in the expression of fusion-related genes (Mfn1, Mfn2) following our treatment suggest a restoration of a functional mitochondrial network through reduced mitochondrial fission and increased mitochondrial fusion. 

 In addition to these mechanisms, it is important to consider the direct effects of treatments on the mitochondria. For example, CoQ_10_, as a component of the electron transport chain, appears to enhance mitochondrial ATP synthesis and reduce the production of ROS (16). Furthermore, transplanted mitochondria may directly fuse with the damaged mitochondrial network and improve the function of this network to enhance ATP synthesis with less ROS production (14). These beneficial improvements collectively reduce the likelihood of ventricular arrhythmia incidence and attenuate IRI in aging. However, additional research is required to gain a comprehensive understanding of the precise mechanisms and signaling pathways that are triggered by these interventions. This necessitates further investigation into the cardioprotective signaling pathways, as well as various proteins, pumps, and channels. Finally, an important aspect of allogenic mitochondrial transplantation is the possibility of potential alloreactivity and immune responses in recipient animals. Although this aspect was not investigated in our study due to the short post-reperfusion observational period, a previous study by Ramirez et al showed that allogeneic or syngeneic mitochondrial transplantation in single or multiple doses did not induce any alloreactivity or immunological responses (42). However, additional study with longer duration of reperfusion is necessary to adequately address this important challenge.

***Limitations***

The techniques used for isolation and purification of mitochondria have been previously verified and accepted in the scientific community, ensuring the quality of the functional mitochondria obtained. Additionally, to track the transplanted mitochondria, we utilized MitoTracker labeling, which stains viable mitochondria, and observed them using fluorescence microscopy. However, the quantification of functionally transferred mitochondria is critical for evaluating therapeutic efficacy. This was not feasible in this research due to certain constraints and should be considered in future investigations. Moreover, ideally, the precision of mitochondrial injections could be improved by considering both the body weight and heart weight of the rats. During the treatment phase, it was not feasible to separate and weigh the heart tissue. In this study, given that the animals were very similar in body weight, a similar number of mitochondria prepared in respiratory buffer was administered to each rat. However, adjusting the number of mitochondria injected based on the body weights of different animals can reduce variability and improve the accuracy of the treatment.

## Conclusion

Co-administration of CoQ_10_ and MT exhibited a more pronounced anti-arrhythmic outcome compared to their individual administration. This was accompanied by a reduction in myocardial injury and an improvement in the histological features of the cardiac tissue. The observed additive effect of these treatments highlights the importance of combination therapies in the development of new therapeutic strategies in the context of IRI in aging. The anti-arrhythmic and protective effects may be in part due to diminishing oxidative stress, increasing NO levels, and restoring the balance between mitochondrial fission and fusion. However, other mechanisms may be involved in the anti-arrhythmic effect of our treatment, which still needs further investigation.

## Data Availability

Data of this study are available from the corresponding author upon reasonable request.

## Declaration

We have not used any AI tools or technologies to prepare this manuscript.
